# Correlation between the morphological features of the biceps groove and injuries to the biceps pulley and the long head tendon of the biceps

**DOI:** 10.1186/s12891-023-06497-5

**Published:** 2023-05-12

**Authors:** Xiaoye Tang, Jialu Zhang, Jiechao Zhang, Yong He

**Affiliations:** Department of Orthopaedic Surgery, Guanghua Hospital affiliated to Shanghai University of Traditional Chinese Medicine, Shanghai, 200052 China

**Keywords:** Biceps groove, Biceps pulley injury, Lesions of LHBT, Three-dimensional reconstruction

## Abstract

**Purpose:**

The morphometric features of the biceps groove were measured to investigate their correlation with the injury of the pulley and the long head of the biceps tendon (LHBT).

**Methods:**

A total of 126 patients undergoing arthroscopic rotator cuff repair surgery had their morphological features of bicipital groove evaluated on a 3D reconstruction model of the humeral head. The groove width, groove depth, opening angle, medial wall angle, and inclination angle of the bicipital groove were measured for each patient. During the surgery, the type of injury to the biceps pulley and the degree of long head of biceps tendon injury were assessed. The correlations of these injury assessments with bicipital groove measurements were analyzed.

**Results:**

The average groove width was(12.3 ± 2.1) mm. The average groove depth was(4.9 ± 1.4) mm. The average groove inclination angle was 26.3° ± 8.1°. The average opening angle was 89.8° ± 18.4°. The average medial groove wall angle was 40.6° ± 7.9°.Sixty six patients had injury of the biceps pulley structure, and their Martetschläger classifications were as follows: type I injury in 12 patients, type II injury in 18 patients, and type III injury in 36 patients. The Lafosse grades of Lesions of LHBT were as follows: 72 cases were grade 0 injury, 30 cases were grade I injury, and 24 cases were grade II injury. We found no significant correlation between the opening width, depth, inclination angle, opening angle, and medial wall angle of the morphological features of bicipital groove and injuries of the pulley and the LHBT. The correlation between pulley structure injury and lesions of LHBT was statistically significant.

**Conclusion:**

Lesions of LHBT show strong correlation with pulley injuries.This study does not find a correlation between the injury of the pulley or the LHBT and bicipital groove morphology.

## Introduction

Lesions of the LHBT are a common source of pain in the shoulder anterior [1–3], and often present concomitantly with other shoulder pathologies. Lafosse et al. reported that 45% of patients with rotator cuff tears also had LHBT lesions [4]. Instability is a common cause, and the biceps groove, the bony channel where LHBT leaves the shoulder joint, is an important stabilizing structure for LHBT [5, 6]. From a morphological point of view, a shallow and wide biceps groove may be a risk factor for LHBT instability and further lesions [5]. Many previous studies have discussed the relationship between biceps groove morphology and LHBT lesions [2, 5, 7], but reports have been inconsistent. Some scholars believe that the internodular groove morphology will affect the stability of the LHBT, thereby causing the damage of the LHBT, but another scholars believe that the damage of LHBT is not related to the internodular groove morphology, and is related to the soft tissue factors above the bicipital groove and subscapularis muscle reconstruction above LHBT. From a methodological perspective, most of the above studies are based on CT or MRI cross-sectional images [8], which cannot guarantee the measurement data because they are easily affected by improper scanning body position and unstable measurement plane selection. The three-dimensional (3D) reconstruction technology of CT bone can superimpose a series of two-dimensional images and reconstruct the three-dimensional structure, allowing for a more intuitive observation of the bone structure from multiple angles [9]. Through the specific fault angle cutting method, an accurate cross-section can be taken for a better view of the biceps groove, which can improve the measurement accuracy and enhance comparability between different individuals.

Before LHBT enters the biceps groove, there exists the long head tendon pulley structure, which is a soft tissue stable structure. The relationship between pulley structure injury and LHBT lesions has attracted increasing attention in recent years [5]. Trauma, degeneration, and intra-articular impingement can all lead to pulley structure injury [10]. As the two important structures that jointly maintain LHBT stability, themorphological features of the inter-tubercular groove of the humerus may also be a risk factor for pulley structure injury. Therefore, it is necessary to perform further research on this.

In this study, patients undergoing arthroscopic shoulder surgery were subjected to a preoperative CT scan, and a 3D model of the proximal humerus was constructed. The width, depth, angle of inclination, angle of opening, and medial wall angle of the biceps groove were measured. Their correlation with pulley structure and LHBT lesions was also investigated.

## Methods

### Study design

This retrospective study was approved by the institutional review board at our institution.The study participant gave written, informed consent to participate in the experiment.Between January 2021 and June 2021, patients who were diagnosed with rotator cuff tear who had undergone MRI and shoulder arthroscopic surgery were selected. During this time frame, there were 142 patients with rotator cuff tears, and 16 patients were excluded, because they had inflammatory diseases, contraindications to surgery, and irreparable rotator cuff tears. A total of 126 patients (54 men and 72 women, mean age 61.05 years) were included. There were 40 cases in the left shoulder and 86 cases in the right shoulder. The patients were 31–78 years old, with an average of 61.05 ± 11.91 years old, and the disease duration was 0.1–3 years, at an average of 1.6 ± 2.26 years.

### Reconstruction of a 3D model of the proximal humerus

The shoulder joint of the patient was CT scanned, with the affected shoulder placed on the side of the body, and a 3D model of the biceps groove was constructed based on CT scan data. A 64-slice CT scanner (SOMATOM Perspective, China) was used, covering the upper end of the humerus. The layer thickness was 0.625 mm, the layer spacing was 0.95 mm, and each pixel of the obtained image was 512 × 512. After scanning, the CT images were preprocessed in CT workstation, and CT data obtained were stored in the DICOM format.

DICOM scan data were imported into Mimics 21.0 software. Threshold analysis was performed using the threshold tool, which was set at 245. The humeral boundary was isolated, the joining images were excised using the zone growth tool, and the excess structures were isolated. The layers were edited one by one, with the remnants replenished and the noise removed. The model was further optimized in Geomagic Warp reverse engineering software. The software was used to fill holes, remove noise, and repair boundaries to smooth the model, resulting in a 3D model of the humeral head containing the biceps groove.

### Positioning the measurement plane

**Fig. 1:**
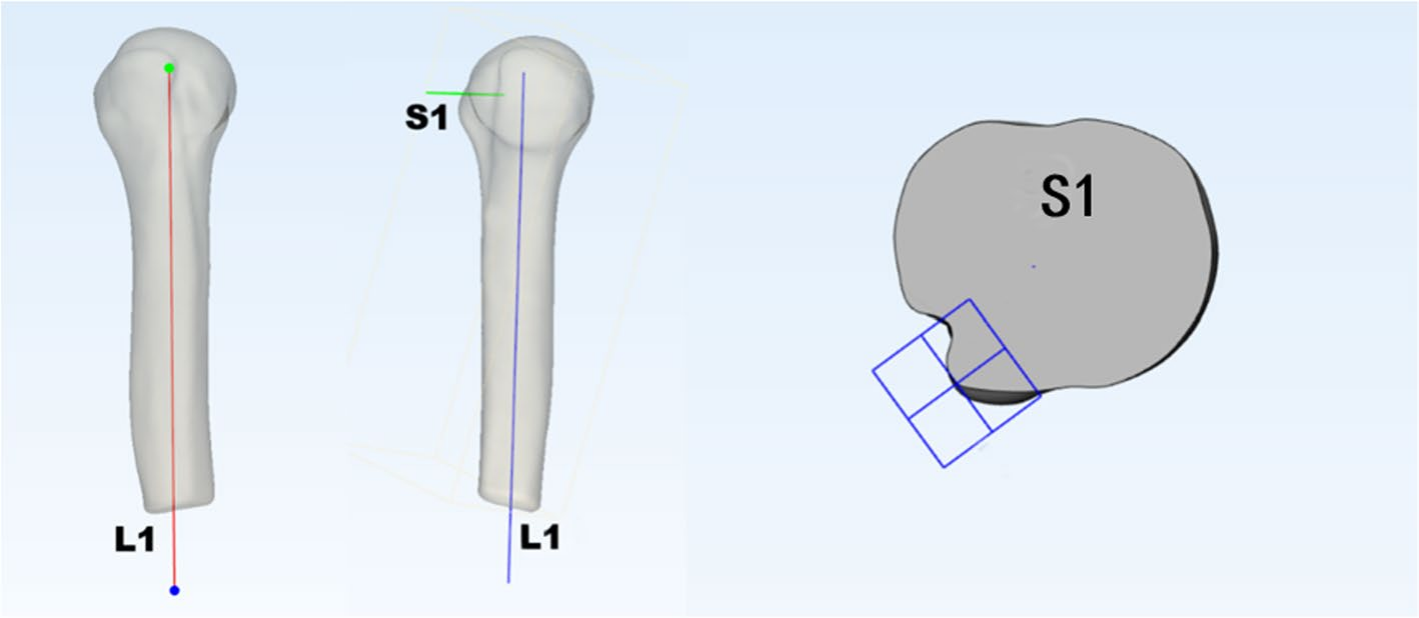
Measurement and positioning

The depth of the biceps groove was defined as the distance from the highest point of the tuberosity to the bottom of the biceps groove [11, 12]. It is critical to locate the measurement plane passing through the highest point of the tuberosity and the bottom of the biceps groove. Previous studies based on CT or MRI data were prone to errors because they only relied on imaging cross-sectional images to select the measurement plane, which could be affected by body position and the angle of administration. In this study, 3-MATIC software was used to set up a humerus model parallel to the humerus shaft Reference line (L1). The highest point of the tuberosity was selected as the reference plane S1 perpendicular to L1. This plane was defined as the measurement plane passing through the highest point of the lesser tubercle and the groove floor, and measurements of the bony parameters of the biceps groove opening were performed on S1 as shown in Fig. 1.

### Measurement of the bony structure of biceps groove

1. Groove width (WG)

On S1 plane, the width of the groove refers to the straight line distance between the vertices of the large and small tuberositys.

(2) Groove depth (DG)

The depth of the biceps groove is the length of a straight line perpendicular to the vertex of the tuberositys.

(3) Opening Angle of biceps groove (OA)

The lowest point of the biceps groove was selected, and a tangent line was made along the lateral wall of the tuberositys. The angle between the two points was the opening angle of the biceps groove.

(4) Medial Wall Angle of biceps groove opening (MWA)

**Fig. 2:**
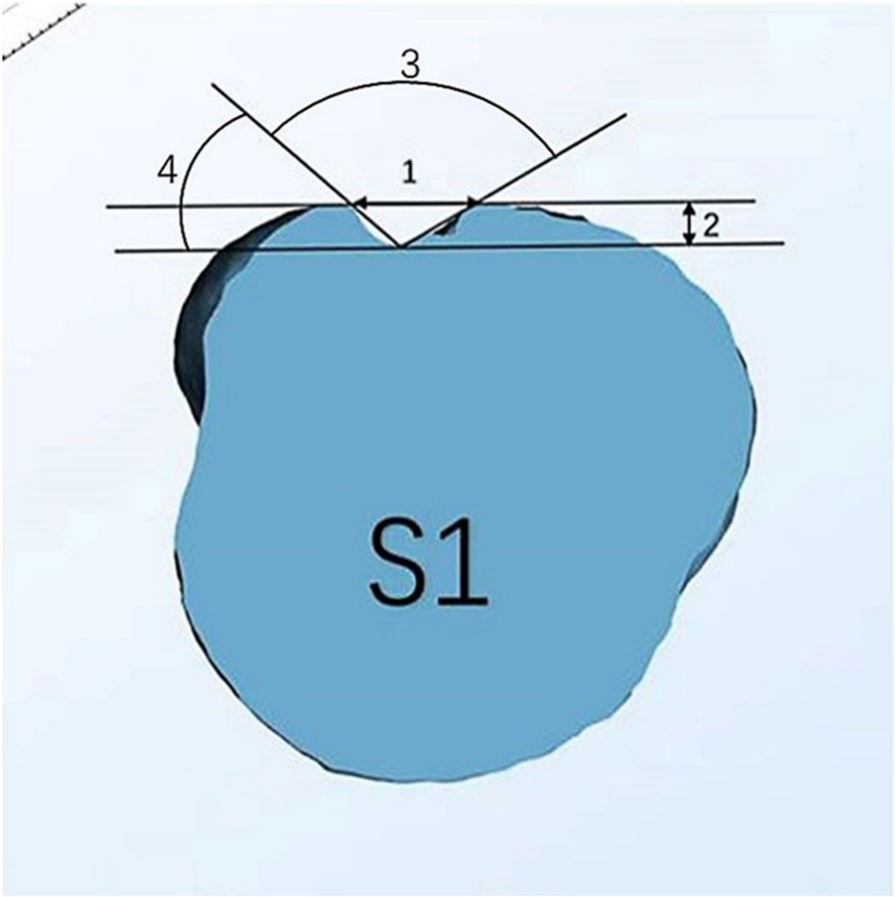
Measurement of morphological features of the biceps groove

MWA refers to the angle between the tangent line passing through the bottom of the intertubercular groove and the medial wall of the intertubercular groove, as depicted in Fig. 2.

Lines are drawn tangential to top and bottom of groove and tangential to medial and lateral border. 1 = groove width; 2 = groove depth;3 = opening Angle of biceps groove;4 = medial Wall Angle of biceps groove opening.

### Measurement of inclination angle of intertubercular groove opening

**Fig. 3:**
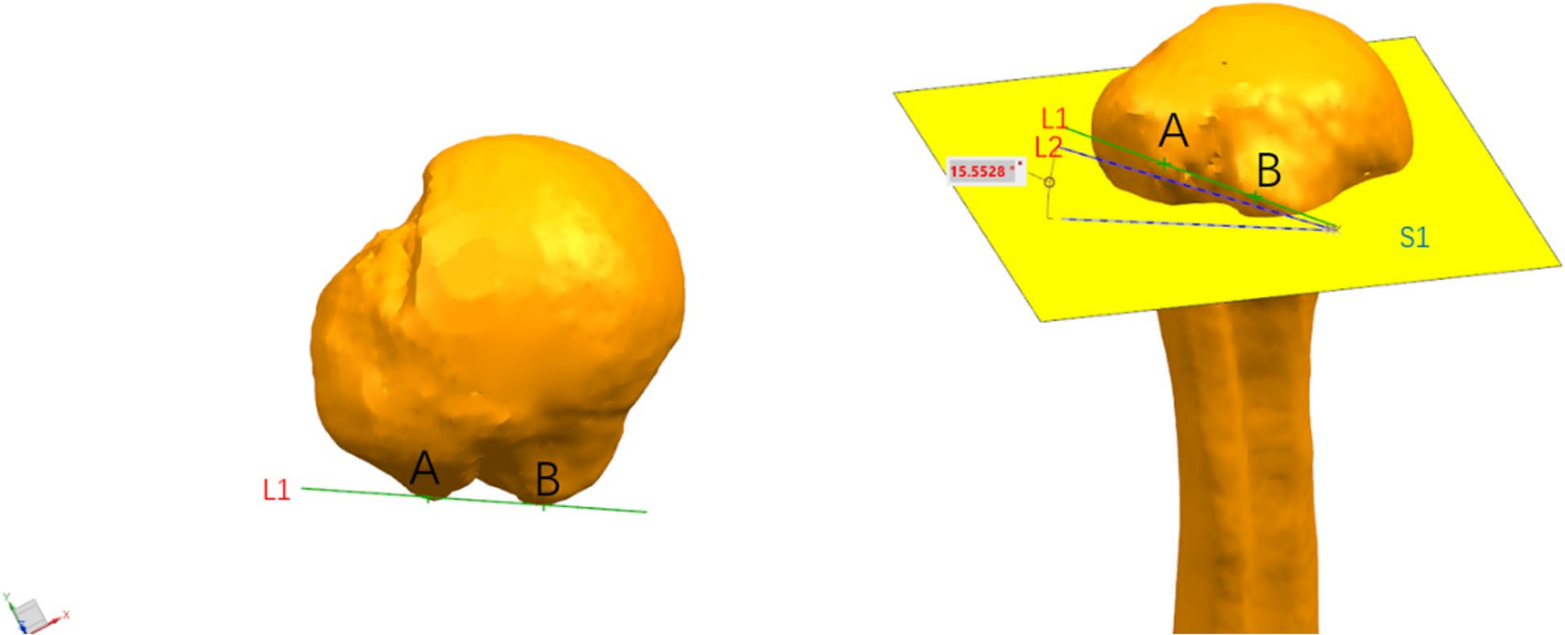
Measurement of the inclination angle of intertubercular groove

In this study, the angle between the two lines of the large and small tuberositys and the transverse line of the humerus shaft was defined as the opening inclination angle of the intertubercular groove. Since the connection of the vertices of the large and small tuberositys is not in the same plane in 3D, the connection L1 of vertices of the large and small tuberositys were directly projected, and the projected line L2 and the vertical segmentation plane S1 of humerus bone were established, in UG (Unigraphics NX Siemens USA) software. The angle between L2 and S1 was measured to obtain the opening inclination angle of the intertubercular groove, as demonstrated in Fig. 3.

### Arthroscopy

Arthroscopy is considered the gold standard for evaluating pulley and Lesions of LHBT. In this study, arthroscopy was performed by the same senior shoulder surgeon to observe the degree of Lesions of LHBT in the glenohumeral joint and classify the pulley structure injury.

1. Classification of pulley structure injury.

**Fig. 4:**
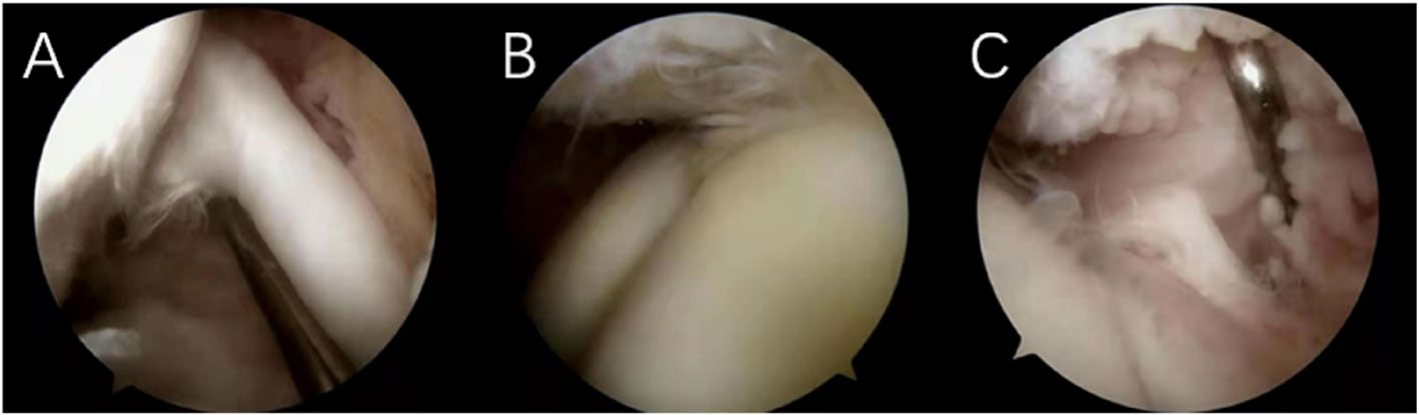
Martetschläger classification of pulley structure injury

The method proposed by Martetschläger [13] was used to classify pulley structure injury, as illustrated in Fig. 4.

- A-Type I:Lesion of the medial pulley (medial coracohumeral ligament and/or superior glenohumeral ligament)
- B-Type II: Lesion of the lateral pulley(lateral coracohumeral ligament)
- C-Type III:Lesion of the medial and lateral pulley slings

2. Classification of Lesions of LHBT

**Fig. 5:**
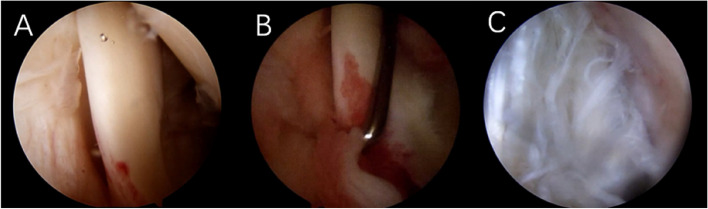
Lafosse grading of biceps longhead tendon injury

The method proposed by Lafosse [4] was used to classify Lesions of LHBT, as shown in Fig. 5.

- A-Grade 0: no injury to long head tendon of biceps
- B-Grade I: minor injury (less than 50% local loss or erosion of tendon)
- C- Grade II: major injury (extensive absence or erosion of more than 50% of the tendon)

### Statistical analysis

The relationship between themorphological features of the biceps groove and injuries to the biceps pulley and the long head tendon of the biceps were analyzedusing the Spearmann correlation test. If data were ordinal and categorical, correlation analysis was performed using gamma test.Statistical analyses were performed using SPSS version 23.0 (SPSS Inc., Chi-cago, IL). *P* values < 0.05 were considered statistically significant.

## Results

### Biceps groove opening morphology

The width of the biceps groove opening ranged from 8.9 mm to 17.2 mm, averaging 12.5 mm ± 2.1 mm. The biceps groove depth ranged from 3.2 mm to 9.2 mm, averaging 4.9 mm ± 1.4 mm. The inclination angle of the biceps groove ranged from 12.7° to 40.4°, averaging 26.3° ± 8.1°. The biceps groove opening angle ranged from 40.9° to 112.7°, averaging 89.8° ± 18.4°. The inner wall angle ranged from 30.9° to 64.3°, averaging 40.6° ± 7.9°.

### Arthroscopy

Sixty patients showed no pulley structure injury, while the remaining 66 patients showed pulley structure injury.According to Martetschläger classification system [13], twelve patients had a type I injury, 18 patients had a type II injury, and 36 patients had a type III injury.

There were 54 patients with LHBT structure injury. According to the Lafosse classification system, 30 patients had a grade I injury and 24 patients had a grade II injury.

### Correlation between pulley structure injury and biceps longus muscle injury

According to the gamma test, the correlation between pulley structure injury and biceps long head muscle injury was statistically significant (*P* < 0.01), with a gamma coefficient of 0.639. These quantities exhibited a positive correlation trend.

### Correlation between morphological features of the biceps groove and lesions of LHBT

All patients were divided into LHBT-injured and non-LHBT-injured groups. After examination, we found no statistical differences in the width, depth, inclination angle, opening angle, and medial wall angle of the biceps groove between the two groups (*P* > 0.05). Table 1 summarizes the result of this examination.

**Table 1:** Comparison of morphological features of the biceps groove between the two groups

	**LHBT-injured group**	**Non-injured group**	***P***
Width of intertubercular groove opening	13.6 ± 2.2	11.8 ± 1.8	0.057
Depth of biceps groove opening	4.9 ± 1.3	4.9 ± 1.6	0.915
Angle of inclination of biceps groove opening	22.6 ± 2.4	28.7 ± 7.2	0.098
Angle of biceps groove opening	90.7 ± 17.4	89.1 ± 19.8	0.855
Medial wall angle of biceps groove opening	42.7 ± 7.7	44.2 ± 8.4	0.694

Spearman correlation test was performed between lesions of LHBT degree and biceps groove morphology, and we found that the correlation between Lesions of LHBT degree and the width, depth, inclination angle, opening angle, and medial wall angle was not statistically significant (*P* > 0.05). Table 2 summarizes the correlation result.

**Table 2:** Correlation between morphological features of the biceps groove and lesions of LHBT grade

Parameter	*r*	*P*
Width of intertubercular groove opening	0.139	0.558
Depth of biceps groove opening	0.054	0.822
Angle of inclination of biceps groove opening	0.296	0.205
Angle of biceps groove opening	0.006	0.979
Medial wall angle of biceps groove opening	0.037	0.876

### Correlation between morphological features of the biceps groove and pulley injury

All 126 patients were divided into the injured pulley structure group and the non-injured group. After examination, there was no statistically significant difference in the width, depth, inclination angle, opening angle, and medial wall angle of the biceps groove between the two groups (*P* > 0.05). Table 3 summarizes the correlation result.

**Table 3:** Comparison of morphological features of the biceps groove between the two groups

	**Pulley injury group**	**Non-injured group**	***P***
Width of intertubercular groove opening	12.4 ± 2.6	12.7 ± 1.6	0.764
Depth of biceps groove opening	5.0 ± 1.8	4.7 ± 0.7	0.620
Angle of inclination of biceps groove opening	26.2 ± 9.3	26.4 ± 6.9	0.955
Angle of biceps groove opening	88.8 ± 20.2	91.1 ± 17.1	0.787
Medial wall angle of biceps groove opening	44.1 ± 9.8	42.9 ± 5.3	0.756

Spearman correlation test was performed between the pulley structure injury type and themorphological features of the biceps groove, and the correlation between the pulley structure injury type and the width, depth, inclination angle, opening angle, and inner wall angle was not statistically significant (*P* > 0.05). Table 4 summarizes the correlation result.

**Table 4:** Correlation between morphological features of the biceps groove and injury degree of pulley structure

Parameter	*r*	*P*
Width of intertubercular groove opening	0.124	0.604
Depth of biceps groove opening	0.042	0.860
Angle of inclination of biceps groove opening	0.202	0.394
Angle of biceps groove opening	0.170	0.472
Medial wall Angle of biceps groove opening	0.033	0.891

## Discussion

In our study, we applied 3D reconstruction methods to measure the intertubercular groove bony morphometric parameters and analyzed their relationships with long head of biceps tendon injuries and pulley structural injuries. There was a positive correlation between the injury of the pulley structure and the lesions of LHBT, but there was no clear correlation between the bmorphological features of the biceps groove and neither the lesions of LHBT nor the pulley injury.

The biceps groove is the most important bony stabilizing structure for LHBT. In this study, average values of the biceps groove was 4.88 mm, while the value from 4.2 to 5.8 mm in other studies [5, 6, 14, 15]. This variation could come from demographic variations in the subjects, such as race, ethnicity, and other factors, or from variations in the measurement method adopted. Previous morphological studies of the biceps groove utilized MRI or CT images of the humerus in cross-section. MRI has natural shortcomings in visualizing bone structure, which may affect the stability of the measurement, especially given that the biceps groove is a long strip that requires a consistent and comparable measurement plane, ideally the humeral cross-section through the highest point of the humeral tuberosity [12]. Obtaining a true cross-section perpendicular to the longitudinal axis of the humerus is difficult due to the differences in position and image scanning parameters during examination. In addition, defining the measurement level in continuous two-dimensional images is difficult, and the determination of the measurement level in previous reports was unclear. According to Joseph et al., [14] MRI biceps groove data from the 4th to 6th layers of the proximal humerus were used, and since they could not guarantee that the same layer was selected, it was difficult to make transverse comparisons between different individuals. Given the above reasons, this study constructed a three-dimensional model of the proximal humerus using thin-slice CT scanning, which allowed us to choose the measurement plane more intuitively and accurately on the model, thus avoiding measurement error and facilitating the horizontal comparison of different studies.

We found no correlation between the bonymorphological features of the internodular groove and the presence of a dolichocephalic tendon lesion.There remains controversy about the correlation between the morphological differences of the biceps groove and LHBT lesions. Yoo et al. [7] compared the intraoperative stability of LHBT with the morphological measurement of the biceps groove based on MRI and found that a shallow biceps groove, large opening angle, and small angle of medial wall were high-risk factors for LHBT instability. Urita et al. [15] used CT data to show that there was no significant correlation between the morphological parameters and LHBT lesions except for the bony spur in the medial wall of the biceps groove and the injury of the subscapularis tendon. Uluckoy et al. [5] also found that, except for subscapular tendinopathy, the shape of biceps sulci had little relationship with LHBT stability. Our study also found no correlation between themorphological features of the biceps groove and Lesions of LHBT, suggesting that the osseous structure is not the only and decisive stabilizing factor of LHBT lesions.

In this study, we found a positive correlation between pulley structure injury and Lesions of LHBT.The pulley structure of LHBT comprises the superior glenohumeral ligament (SGHL), the coracohumeral ligament (CHL), the supraspinatus muscle, and the subscapularis tendon, which is a soft tissue stabilizing device before the biceps long head tendon enters the bone pulley groove. Pulley structure injury is an important reason for the anterior pain of the shoulder joint [16–18]. Recently, more attention has been paid to pulley injury. Habermeyer classification is a commonly used clinical injury classification system for pulley structures [15]. This system was innovated by Martetschläger et al. [13], and it was accurate, easy to use, and has a short processing time. In the present study, the proportion of pulley structure injury reached 55%, which was much higher than the 7% reported by Baumann [5]. This may be because this study enrolled more patients who required rotator cuff repair, whereas Baumann included a larger number of diagnostic arthroscopy cases. Considering that the biceps groove and the trochlea structure are the bone and soft tissue stabilizing structures of LHBT respectively, they may influence each other in the development of LHBT lesions.

This study has many shortcomings. All patients included in this study had rotator cuff tears, shoulder impingement, or other serious cases requiring surgery, and these diseases are high-risk factors of LHBT and pulley injuries [18] and could interfere with the analysis results. Second, due to the limited workload and research time of 3D reconstruction, this study could only include 126 cases, which may affect the validity of the conclusions. Finally, the accuracy of the study and the comparability between individuals were improved by positioning and measurement on the 3D model. However, most of the measurement parameters used for comparison were still measured in 2D studies. And, the measurement plane of this study was through greater tuberosity only, ignoring the influence of lesser tuberosity.Further exploration is required to verify the advantages of 3D modeling in studying the biceps groove.

In conclusion, there are variations in the morphological features of bicipital groove, but the correlation between the pulley structure and Lesions of LHBT was not found in this study.

## Data Availability

The datasets generated and/or analyzed during the current study are not publicly available but are available from the corresponding author on reasonable request.
